# A Comparison among Lignin Modification Methods on the Properties of Lignin–Phenol–Formaldehyde Resin as Wood Adhesive

**DOI:** 10.3390/polym13203502

**Published:** 2021-10-12

**Authors:** Hamed Younesi-Kordkheili, Antonio Pizzi

**Affiliations:** 1 Department of Wood and Paper Sciences and Technology, Faculty of Natural Resources, Semnan University, Semnan 35131-19111, Iran; 2 LERMAB-ENSTIB, University of Lorraine, 27 rue Philippe Seguin, 88000 Epinal, France

**Keywords:** phenol–formaldehyde, lignin modification, maleic anhydride, ionic liquid, glyoxalation

## Abstract

The research aim of this work is to determine the influence of lignin modification methods on lignin–phenol–formaldehyde (LPF) adhesive properties. Thus, glyoxal (G), phenol (P), ionic liquid (IL), and maleic anhydride (MA) were used to modify lignin. The modified lignins were used for phenol substitution (50 wt%) in phenol–formaldehyde adhesives. The prepared resins were then used for the preparation of wood particleboard. These LPF resins were characterized physicochemically, namely by using standard methods to determine gel time, solids content, density, and viscosity, thus the physicochemical properties of the LPF resins synthesized. The panels dimensional stability, formaldehyde emission, bending modulus, bending strength, and internal bond (IB) strength were also measured. MA-modified lignin showed by differential scanning calorimetry (DSC) the lowest temperature of curing than the resins with non-modified lignin and modified with IL, phenolared lignin, and glyoxal. LPF resins with lignin treated with maleic anhydride presented a shorter gel time, higher viscosity, and solids content than the resins with other lignin modifications. Equally, the particleboard panels prepared with LPF resins with maleic anhydride or with ionic liquid had the lowest formaldehyde emission and the highest mechanical strength among all the synthesized resins. The dimensional stability of all panels bonded with modified lignin LPF resins presented no difference of any significance.

## 1. Introduction

Phenol–formaldehyde (PF) resins are used to bind moisture and water-resistant wood-based panels such as particleboard, plywood, and oriented strandboard (OSB), thus giving stability to temperature and preventing panels from delaminating [1,2]. However, PF resins are expensive due to the price of phenol and present formaldehyde toxicity. Thus, research aimed at using lower-cost biomaterials as phenol substitutes in PF resins is increasing. One of the current proposals on this subject is to substitute phenol with natural renewable materials such as tannin, cornstarch, etc. [3]. In this regard, previous work indicated that lignin, due to its chemical similarity with phenol, showed the best results among all-natural materials [4]. Perez et al.indicated that phenol could be in part substituted by lignin, attaining the specifications for its final applications [5]. Lignin can be obtained from different sources, but the one obtained from black liquor is the favorite due to its abundance, lower cost, and being environment-friendly [6]. However, the main obstacle to increasing lignin application to phenolic adhesives is due to its slower reaction than phenol with formaldehyde. For this reason, it is proven that lignin must be modified before any use of it in the PF resin for wood adhesive. Some lignin pretreatments have shown to be capable of overcoming, in part, the slower reaction with formaldehyde. Thus far, several methods have been proposed for lignin modification, such as ionic liquids treatment, glyoxalation, reduction, phenolation, etc. [7]. The LPF resin characteristics can be influenced by the different types of chemical materials used and the modification processes of lignin. Hence, this work aims to compare the best proposed modification methods of lignin for use in a PF resin.

Several research works have been focused so far on how lignin types influence the properties of the PF resin [8]. LPF resins properties derived by the use of modified lignins have also been presented is some works [9,10]. As a consequence, this study highlights a number of lignin modification methods for substituting phenol in LPF adhesives.

## 2. Materials and Methods

### 2.1. Materials

The pH 13 soda bagasse black liquor was obtained from the Pars Company (Haft Tepe, Iran). Lignin extraction from the black liquor was done by acidifying with sulfuric acid. Maleic anhydride, Glyoxal, phenol, 1-ethyl-3-methylimidazolium acetate ([Emim][OAc]), an ionic liquid, and other chemical materials used were acquired from Merck Co. (Kenilworth, NJ, USA).

### 2.2. Methods

#### 2.2.1. Lignin Modifications

1. Glyoxalation of Lignin

Lignin was glyoxalated by the procedure described in Younesi-Kordkheili et. al [10].

2. Phenolation of Lignin

Lignin was phenolated by the procedure described in Younesi-Kordkheili et al. [11].

3. Ionic Liquid Treatment

After mixing lignin with [Emim][OAc] ionic liquid at a ratio of 1:20, a 5:1 addition of deionized water was used to then filter and recover the material after a 30 min reaction at 120 °C. The recovered solids were first washed then dried overnight at 40 °C under vacuum.

4. Maleic Anhydride Treatments

DMSO was used to dissolve sufficient lignin in a glass reactor equipped with reflux condenser and mechanical stirring. Then, 400 µL 1-methylimidazole was added dropwise under continuous stirring to catalyze lignin esterification, immediately followed by 40 g maleic anhydride. The mixture was then heated to 80 °C under continuous mechanical stirring for 3 h. After cooling, the maleated lignin was then recovered by precipitating it at pH 3. The unreacted maleic anhydride was then eliminated by continuous washing with water. Finally, the residual solids were oven-dried at 60 °C.

#### 2.2.2. Synthesis of LPF Resin

The synthesis was done as described in the procedure in Younesi-Kordkeili et al. [11].

### 2.3. Physicochemical Properties

Gel time, viscosity, solids content, and density of the synthesized resins were determined in triplicate by using standard methods and averaged.

### 2.4. Fourier Transform Infrared Spectrometry (FTIR)

The functional groups and chemical characteristics of lignin before and after modifications were obtained using a Fourier Transform Infrared Spectroscopy (IRAFFINITY-1, Shimadzu, Kyoto, Japan) with a resolution of 4000–400 cm^−1^ using 20 scans per sample using KBr pills containing 1% by weight of resin powder.

### 2.5. Differential Scanning Calorimetry Analysis

A NETZSCH DSC 200 F3 Model thermal analyzer was used to determine the curing temperature changes of the LPF resins prepared. A heating rate of 10 °C/min under nitrogen atmosphere at a flow rate of 60 mL/min flow rate were the conditions used for the DSC scans. Two replicates per resin were used for DSC analysis.

### 2.6. Panel Manufacturing

35 × 35 mm triplicate laboratory particleboards of 16 mm thickness and aimed density of 0.700 g/cm^3^ were prepared according to previous work of Younesi-Kordkheili et al. 2016 [10] at 10% solids resin load, 170 °C hot press temperature, and 25 bar maximum pressure, for 5 min press time. The panels were tested after 48 h stacking.

### 2.7. Properties of Panels

The particleboards prepared were tested according to standard methods. The internal bond (IB) strength and static bending (bending modulus and bending strength) were tested according to EN-319 and EN-310, respectively. Water absorption and thickness swelling of the panels were measured according to ASTM- 4442. The subsequent formaldehyde emission from the particleboard panels was done using the flask method according to EN 717-3. The samples were conditioned at a temperature of 23 °C ± 2 °C and a relative humidity of 60% ± 5% for two weeks before any testing. Five specimens were tested for each panel.

### 2.8. Statistical Analysis

Data for each test were statistically analyzed. The effects of unmodified and modified lignin content on the panels’ properties were evaluated by analysis of variance (ANOVA) at a 95% confidence level. When the ANOVA indicated significant differences between the factors, the compared values were evaluated with the Tukey Test.

## 3. Results and Discussion

### 3.1. Physicochemical Properties

The properties of the resins are shown in Table 1. This shows that the modified LPF adhesive had higher solids content, higher viscosity, density, and a shorter gel time than the resins made from unmodified lignin and modified by the other three treatments. The shorter gel time of the maleated LPF resin is likely due to the higher reactivity induced in lignin sites by maleation. It may well be due, quite likely, to a greater extent of reaction and increased crosslinking between the two materials. Previous research has already shown that by including in a phenolic resin, modified lignin increases resin viscosity and renders the gel time faster [11,12]. Based on the physicochemical test analysis results, the resins modified by maleic anhydride and ionic liquid treated lignin had higher solids of all the resins synthesized. Thus, the larger increase in viscosity of the maleated LPF resin and of the LPF resin with ionic liquid-treated lignin is likely to be due to both chemical effects related to an increased level of crosslinking and to physical effects due to the higher resin solids content. The results of these tests show that the phenolated lignin LPF resin has the lowest density (1.222), while the maleated LPF resin had the highest density (1.228).

### 3.2. FTIR Analysis

**Figure 1:**
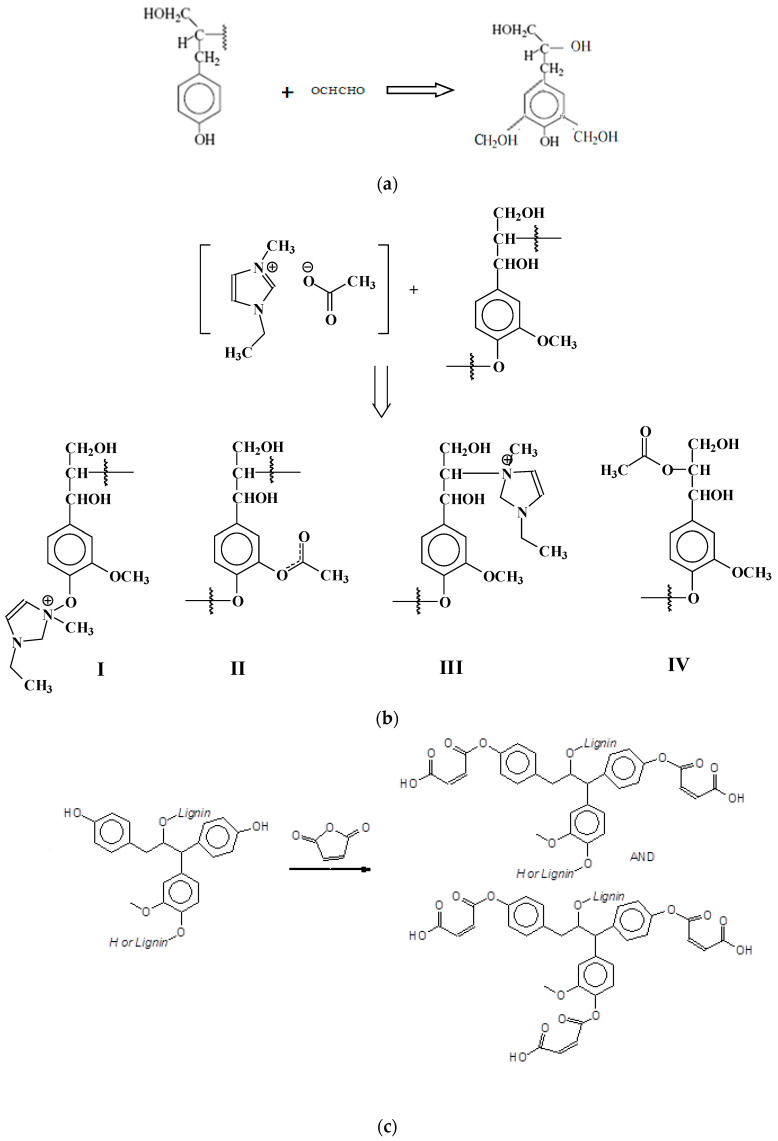

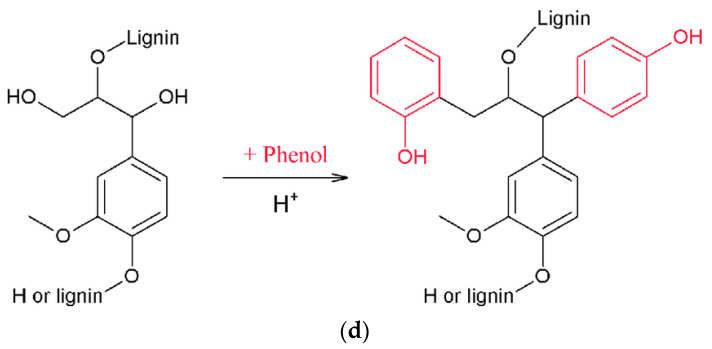
Schematic reactions between lignin and (**a**) glyoxal, (**b**)ionic liquid, (**c**) maleic anhydride, and (**d**) phenolation.

**Figure 2:**
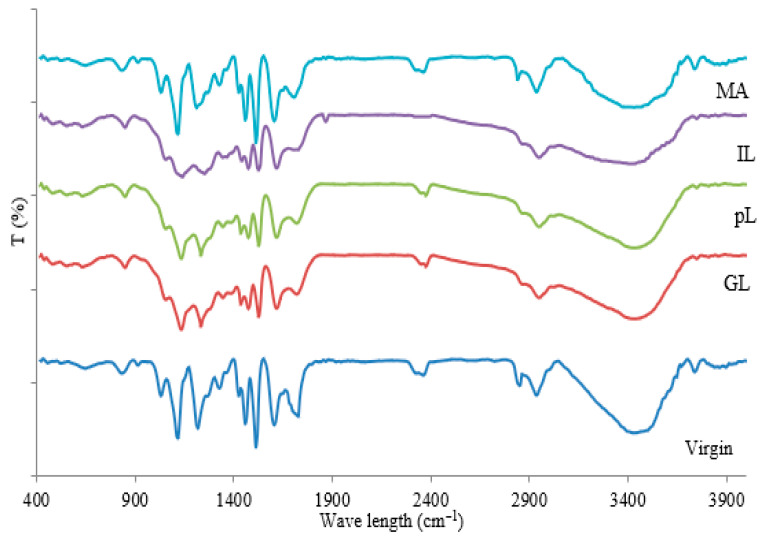
FTIR analysis of pure and modified lignin.

The Characteristic reactions of the lignin modifications (Figure 1) and the infrared spectra of the modified and control lignins are shown in Figure 2. When comparing the infrared spectra of the various lignins, one notices in maleated lignin the variation of a few main peaks. When comparing the infrared spectra of maleated lignin to the unmodified one in the maleated lignin, the intensities of the 1700 cm^−1^ and 2800 cm^−1^ bands respectively assigned to COOH and C-O groups increase. The band at 1700 cm^−1^ is particularly indicative of the presence of esters, showing that maleic anhydride has definitely reacted with and esterified the lignin and is characteristic of coordinated unsaturated esters confirming the configuration shown in the schematic Figure 2 for maleated lignin. Furthermore, the intensity of the 1200 cm^−1^ band assigned to the C=C bonds of maleated lignin increased when compared to pure lignin. It is interesting to note that the bands at 1600, 1300, and 970 cm^−1^ confirm that the configuration around the C=C double bond is trans. Furthermore, the lignin modified with the maleic anhydride showed a smaller peak at 3420 cm^−1^ (the hydroxyl group) than the neat lignin, this being due to the esterification reaction. Figure 2 shows that the peak at 3440 cm^−1^ decreases markedly after ionic liquid lignin modification. This band is assigned to phenolic and aliphatic hydroxyl groups (-OH) stretching. The IL modified lignin showed a more intense 1685 cm^−1^ peak, assigned to C=O stretching, and a 1215 cm^−1^ peak assigned to C-C and C-H bond than other modified lignins. The formation of C-N bonds of IL with lignin is indicated by the new peak at 1852 cm^−1^ (Figure 2). The O-H stretching peak at 3420 cm^−1^ clearly increases in phenolated lignin. This effect is due to the significant increase in phenolic –OHs caused by phenolation. Compared to unmodified lignin, the 1330 cm^−1^ band related to the aliphatic ethers C-O bond assigned to the 1330 cm^−1^ peak markedly decreases in the modified resin when compared to the unmodified lignin. Glyoxalation logically induces in lignin an increase of the 3440 cm^−1^ peaks assigned to phenolic and aliphatic -OH stretching. Lignin glyoxalation also causes a significant increase of C=O groups when compared tounmodified lignin.

The possible reactions between lignin and glyoxal, ionic liquid, phenol, and maleic anhydride are shown in Figure 1.

### 3.3. DSC Analysis

**Figure 3:**
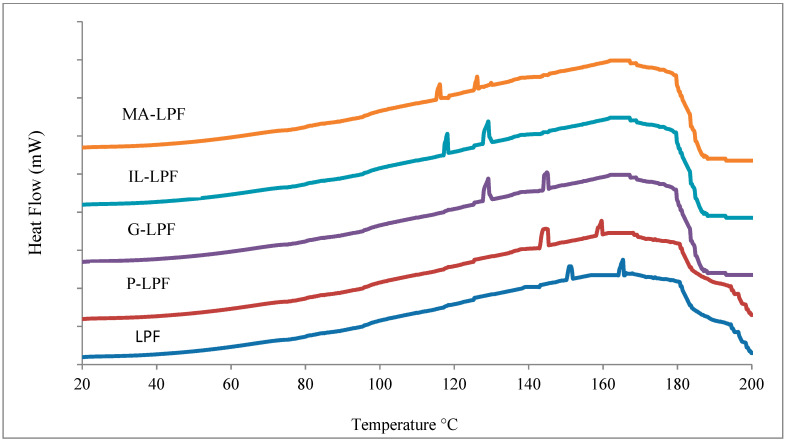
DSC analysis of synthesized LPF resin.

Figure 3 reports the DSC thermographs of the modified and unmodified LPF resins. Resin curing appears to occur in two steps, as shown by the presence of two exothermal peaks in all the modified and unmodified LPF resins DSC curves. For example, the two exothermal peaks occur at 142 °C and 169 °C for the unmodified LPF control resin. The modified LPF resins show a progressive decrease in the temperatures of the two exothermal peaks as one passes from the unmodified to the phenolated LPF to the maleate LPF resin, this latter presenting the two exotherms at the lowest temperatures of all the other resins (Figure 3). Gel times values support these results as they are shortened as modified lignins are included in the LPF resins. The maleated LPF resin fast curing confirms that including maleated lignin shortens the phenolic resin curing, this being partially due to its high crosslinks content in comparison with other types of modified lignin and the unmodified LPF resin. Other reasons explained later also contribute to such an acceleration effect. Several different research groups have indicated that modified LPF resin cures at lower temperatures and faster than a PF resin [13,14,15,16,17,18,19,20,21,22,23,24,25,26,27,28].

### 3.4. Properties of Panels

The flexural modulus and flexural strength of the panels prepared are shown in Table 2. Flexural analysis indicates that greater flexural modulus and flexural strength can be achieved by modification of lignin. The samples bonded with an LPF resin with maleated lignin present higher flexural modulus and flexural strength than those bonded with LPF resins prepared with other types of modification. The lowest flexural modulus (2510 MPa) and strength (17 MPa) were shown by the panels with unmodified lignin LPF adhesive. Instead, the maleated LPF-bonded panels presented the highest flexural modulus (2953 MPa) and strength (25 MPa) values, respectively. The higher flexural modulus and strength of the panels bonded with LPF resins with maleated lignin can be probably attributed to the greater stiffness and strength of the bonds between reactive sites of the maleated lignin chains and formaldehyde and phenol can partially account for its better performance [29].

Younesi-Kordkheili and Pizzi, have shown that a better resin polymerization results in improved flexural properties of the panels [30]. The internal bond (IB) strengths of the particleboards prepared with the LPF resins are shown in Table 2. As shown in Table 2, the incorporation of maleated lignin into PF resin yielded the highest IB strength. Thus, while the highest IB strength is shown by the panels bonded with the LPF resin with maleated lignin (0.76 MPa), the lowest IB strength (0.65 MPa) is shown by the panels bonded with the LPF resin with unmodified lignin. Generally, the panels made from M-LPF, IL-LPF, G-LPF, and P-LPF resins exhibited 5%, 6%, 10%, and 17% higher IB strength than those one made from unmodified LPF resin, respectively. The highest IB strength of the panels bonded with maleated lignin LPF adhesives compared to those with LPF resins with other types of lignin modifications can be related to the difference in the type and the amount of chemical bonds and to the physicochemical quality of the resins. Additionally, the effect of maleation of lignin causes the formation of maleic esters on the free hydroxyl groups of lignin, both aromatic and aliphatic. Esters of any type cause acceleration of the reactions of formation and curing of phenol–formaldehyde resins. The effect was first discovered in the late 1950s and applied for PF resins for binding foundry core sand molds [31,32], where 50% of the PF resin was the ester added (in general methyl formiate). It was subsequently extensively modified for application to PF adhesives for wood panels bonding [33,34], in particular with the considerable decrease of the percentage of ester accelerator, between 3% and 5% of the PF resins solids, and the use of more effective ester accelerators for wood application, such as propylene carbonate and in particular glycerol triacetate (triacetine). It is now a well-known effect and has been studied in depth [35]. It is applicable both as an alpha-set (esters in vapour phase) or beta-set (esters added in liquid form to the PF resin). Lei et al. 2006 determined by solid-state CP MAS 13C NMR and MALDI ToF mass spectrometry that the ester caused additional crosslinking of the resin by forming additional and chemically different bridges than the normal –CH2- ones between the phenolic aromatic nuclei. Additionally, it reacted and crosslinked the phenol normally unreactive meta sites of aromatic rings and was different as the bridges have the nature of ketones [36]. The final network is thus formed more rapidly due to the additional reaction and is thus much more crosslinked and hence much stronger. In the research work presented here, lignin has been esterified; thus, the maleated lignin assumes two fundamentally new functions on top of the one as phenol substitute: that of an accelerator forming additional ketone-like bridges between aromatic nuclei meta sites of both the phenol of the PF resin and the aromatic rings of the lignin itself. Furthermore, the role of a crosslinker increased the network tightness and strength; hence, the higher IB strength of the maleated LPF was found.

**Figure 4:**
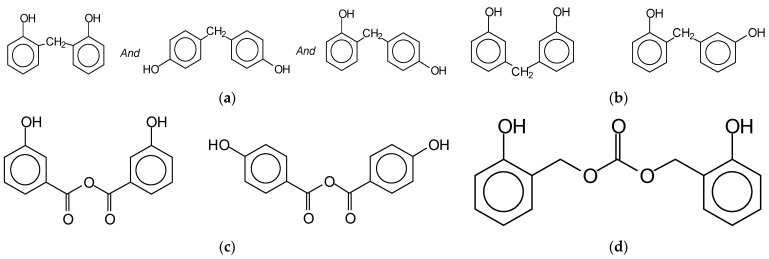
Different bridges between the maleated lignin and PF resin.

**Figure 5:**
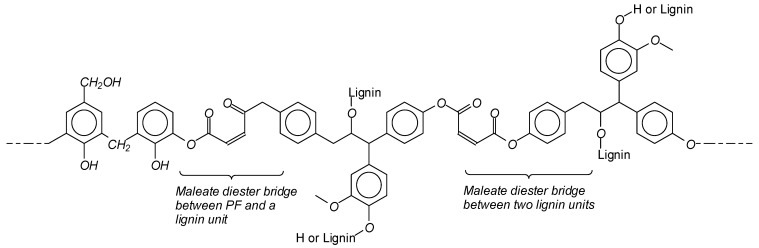
Schematic interaction between maleated lignin and the PF resin.

The reaction of preparation of the maleated lignin can then be represented as in Figure 1. The maleated lignin accelerator induces in the PF resins not only the classic –CH_2_- type of bridges (Figure 4a) but also additional intermediate bridges such as m,m-dihydroxydiphenyl methane and m,o-dihydroxydiphenyl methane ones (Figure 4b) and intermediate temporary anhydride structures (Figure 4c,d) [35]. Moreover, as on opening the maleic anhydride ring, the two carboxylic acid functions of maleic acid are generated, maleate crosslinking bridges between lignin and the PF resin as well as between two lignin units on different chains can probably also be formed, further contributing to the final network strength, such as indicated in Figure 5.

Water absorption and thickness swelling of the panels made from LPF resin with modified lignins are shown in Table 2. The LPF resin with phenolated lignin presented the lowest water absorption (41%) and thickness swelling (16%), while the unmodified LPF resin had the highest water absorption (52%) and thickness swelling (20%). It can also be seen that there are no significant differences among water absorption and thickness swelling of the panels made from modified LPF resins. Previous research indicated that crosslinked phenolic structures are hydrophobic [36]. Conversely, panels bonded with phenolated LPF adhesives presented a slightly reduced water absorption and thickness swelling than those bonded with unmodified lignin LPFs. This is due to the increase of the lignin reactive sites by the grafting of phenol on it, inducing greater reactivity and crosslinking. That modification of lignin improves the LPF-bonded panels’ dimensional stability has already been reported by several researchers [37].

**Figure 6:**
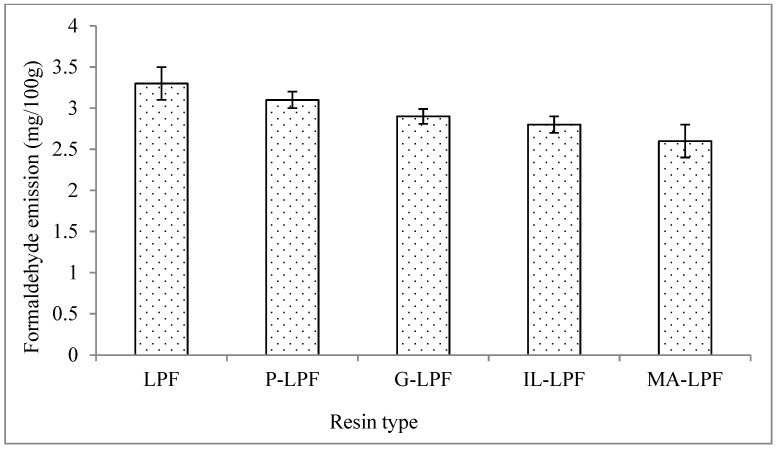
Formaldehyde emission of the prepared panels.

Panels bonded with LPF resins with all types of modified lignins had lower subsequent formaldehyde emissions compared to those bonded with unmodified resins (Figure 6).

This may be explained by the fact that bridges involving completely or partially phenolic moieties such as modified lignin to formaldehyde are stable, while the same bridges connecting unmodified lignin to phenol may not be as stable and hence susceptible to hydrolysis then releasing formaldehyde. For these reasons, the modified lignin-based resins yield lower formaldehyde emissions compared to unmodified LPF resins. Lower formaldehyde emission has been reported for particleboard by addition of modified lignin into phenol formaldehyde (PF) resin by Mu et al. (2009), notwithstanding the fact that modified LPF bonded boards usually show already a very low subsequent formaldehyde emission [38]. Figure 6 also indicates that the panels bonded with maleated lignin presented the lowest formaldehyde emission (2.6 mg/100 g) among the ones with all modified lignins. The higher level of crosslinking of the maleated LPF resin explains its bonded panels lower formaldehyde emission, hence as a consequence of the higher bridges proportion per unit volume than the other types of modification methods. This is also due to a part of the free formaldehyde available in the solution that can react with the active sites of lignin during maleated LPF resin synthesis, resulting in a lower formaldehyde emission from the panels.

## 4. Conclusions

Nowadays, the use of lignin as a substitute for phenol in PF resins is growing. The aim of this work was to evaluate the effect of lignin modification methods on the properties of lignin–phenol–formaldehyde (LPF) resins as a wood adhesive. The conclusions are then as follows.

Using any form of modified lignin as a substitute for phenol effectively improves the characteristics and performance of aphenolic resin.

The cure temperature of the resin decreases with all the modification treatments of lignin was confirmed by the DSC results. However, the maleated lignin LPF resin had the lowest cure temperature among all the lignin modification methods tested.

The highest viscosity and shortest gel times were presented by the maleated lignin LPF resin.

The lowest formaldehyde emission and the highest mechanical strength were shown by the particleboards bonded with maleated lignin LPF and ionic liquid modified LPF resins.

There were no obvious differences in dimensional stability of the panels bonded with LPF resins modified with all types of modification.

## Figures and Tables

**Table 1:** Physicochemical properties of LPF resins.

Resin	Density (g/cm3)	Gel time (S)	Viscosity (cP)	Solid Contents (%)
LPF	1.221 ^c^	357 ^a^	342 ^d^	55 ^c^
P-LPF	1.222 ^c^	325 ^b^	377 ^c^	56 ^c^
G-LPF	1.223 ^c^	311 ^c^	396 ^b^	58 ^b^
IL-LPF	1.225 ^b^	293 ^d^	421 ^ab^	61 ^a^
MA-LPF	1.228 ^a^	288 ^e^	430 ^a^	61 ^a^

Means with different letters within the column are significantly different (*p* < 0.05).

**Table 2:** The properties of the panels made from synthesized resins.

Adhesive Type	Flexural Modulus (MPa)	Flexural Strength (MPa)	IB Strength (MPa)	Water Absorption (%)	Thickness Swelling (%)
LPF	2510 ^d^ ± 80	17 ^c^ ± 2.1	0.65 ^d^ ± 0.01	52 ^a^ ± 3.2	20 ^a^ ± 0.7
P-LPF	2644 ^c^ ± 91	19 ^bc^ ± 1.2	0.68 ^cd^ ± 0.02	41 ^d^ ± 1.3	16 ^d^ ± 0.3
G-LPF	2811 ^b^ ± 36	20 ^b^ ± 1.4	0.69 ^c^ ± 0.03	46 ^b^ ± 1.3	18 ^b^ ± 0.3
IL-LPF	2832 ^ab^ ± 100	22 ^ab^ ± 1.3	0.72 ^b^ ± 0.02	43 ^c^ ± 1.2	17 ^c^ ± 0.1
MA-LPF	2953 ^a^ ± 96	25 ^a^ ± 1.1	0.76 ^a^ ± 0.01	44 ^c^ ± 1.1	17 ^c^ ± 0.2

Means with different letters within the column are significantly different (*p* < 0.05).

## Data Availability

Not applicable.
